# Frontal Theta Oscillations in Perceptual Decision-Making Reflect Cognitive Control and Confidence

**DOI:** 10.3390/brainsci16020123

**Published:** 2026-01-23

**Authors:** Rashmi Parajuli, Eleanor Flynn, Mukesh Dhamala

**Affiliations:** 1 Department of Physics and Astronomy, Georgia State University, Atlanta, GA 30303, USA; 2 Neuroscience Institute, Georgia State University, Atlanta, GA 30303, USA; 3 Center for Behavioral Neuroscience, Georgia State University, Atlanta, GA 30303, USA; 4 Center for Diagnostics and Therapeutics, Georgia State University, Atlanta, GA 30303, USA; 5 Tri-Institutional Center for Translational Research in Neuroimaging and Data Science (TReNDS), Georgia State University, Georgia Institute of Technology, and Emory University, Atlanta, GA 30303, USA

**Keywords:** perceptual decision-making, theta oscillations, EEG, stimulus clarity, current source density, cognitive control, functional connectivity, imaginary coherence

## Abstract

Background: Perceptual decision-making requires transforming sensory inputs into goal-directed actions under uncertainty. Neural oscillations in the theta band (3–7 Hz), particularly within frontal regions, have been implicated in cognitive control and decision confidence. However, whether changes in theta oscillations reflect greater effort during ambiguous decisions or more efficient control during clear conditions remains debated, and theta’s relationship to stimulus clarity is incompletely understood. Purpose: This study’s purpose was to examine how task difficulty modulates theta activity and how theta dynamics evolve across the decision-making process using two complementary analytical approaches. Methods: Electroencephalography (EEG) data were acquired from 26 healthy adults performing a face/house categorization task with images containing three levels of scrambled phase and Gaussian noise: clear (0%), moderate (40%), and high (55%). Theta dynamics were assessed from current source density (CSD) time courses of event-related potentials (ERPs) and single-trials. Statistical comparisons used Wilcoxon signed-rank tests with false discovery rate (FDR) correction for multiple comparisons. Results: Frontal theta power was greater for clear than noisy face stimuli (corrected *p* < 0.001), suggesting that theta activity reflects cognitive control effectiveness and decision confidence rather than processing difficulty. Connectivity decomposition revealed that frontoparietal theta coupling was modulated by stimulus clarity through both phase-locked (evoked: corrected *p* = 0.0085, dz = −0.61) and ongoing (induced: corrected *p* = 0.049, dz = −0.36) synchronization, with phase-locked coordination dominating the effect and showing opposite directionality to the induced components. Conclusions: Theta oscillations support perceptual decision-making through stimulus clarity modulation of both phase-locked and ongoing synchronization, with evoked component dominating. These findings underscore the importance of methodological choices in EEG-based connectivity research, as different analytical approaches capture different aspects of the same neural dynamics. The pattern of stronger theta activity for clear stimuli is consistent with neural processes related to decision confidence, though confidence was not measured behaviorally.

## 1. Introduction

Perceptual decision-making is the cognitive process by which sensory information is gathered, integrated, and transformed into goal-directed behavior [1]. This process even under noisy conditions allows individuals to navigate complex environments by transforming ambiguous sensory inputs into purposeful actions [2]. Unlike simple stimulus recognition, perceptual decision-making involves accumulating evidence over time and resolving uncertainty before committing to a choice [3,4]. Understanding the neural mechanisms that underlie this transformation from perception to action is essential particularly when decisions must be made rapidly or under conditions of degraded sensory input [5,6].

The computational demands of perceptual decision-making become particularly evident when sensory information is degraded or ambiguous [7,8]. This process involves complex interactions between sensory processing areas and higher-order cognitive control regions, with the efficiency of these interactions determining both the speed and accuracy of decisions [9]. The neural mechanisms supporting these interactions have become a central focus of cognitive neuroscience research, as they provide insights into how the brain adapts to varying environmental demands [10].

Neural oscillations, rhythmic electrical activities generated spontaneously or in response to stimuli in the central nervous system, are vital to sensory-cognitive functions [11,12]. These oscillations serve as a fundamental mechanism for coordinating neural activity across distributed brain networks, enabling efficient information processing and communication between brain regions [13,14]. Among these oscillatory patterns, the theta rhythm is particularly relevant to behavior and cognition [15,16]. Theta oscillations, prominent in the hippocampus of rodents during voluntary behaviors, also appear across multiple human brain regions and are implicated in attention and memory processes [17,18]. They are especially significant in distributed brain networks beyond the hippocampus [11,19,20]. While neural oscillations have been extensively studied across various cognitive domains, their relationships to stimulus degradation during perceptual decision making remain incompletely characterized. Prior work examining degraded visual stimuli has primarily focused on object recognition and perceptual binding, with the most attention directed toward gamma and alpha band activity rather than theta oscillations [21,22]. Studies manipulating visual noise have demonstrated effects on early sensory processing and perceptual thresholds, but the relationship between perceptual clarity and frontal theta during explicit decision-making tasks has not been systematically investigated. Critically, most exciting theta research has manipulated cognitive conflict or general task difficulty through response competition paradigm rather than parametrically varying perceptual evidence quality through stimulus degradation, leaving uncertain whether frontal theta responses reflect increased processing effort or more fundamentally track the quality of available sensory evidence.

The functional significance of theta oscillations extends across multiple cognitive domains, including working memory maintenance, attention allocation, and cognitive control [23,24]. During demanding cognitive tasks, theta oscillations appear to synchronize activity across frontal and parietal brain regions, facilitating the integration of information necessary for complex decision-making [19,25]. This synchronization may serve as a temporal framework that coordinates the timing of neural processing across different brain areas, ensuring that relevant information is available when needed for decision formation [26].

Recent studies have emphasized the role of theta oscillations in perceptual decision-making, particularly within frontal and parietal cortical networks. Frontal theta power reliably increases with task engagement and cognitive demand [27] and is associated with higher-order cognitive functions including conflict monitoring, error detection, and executive control [25,28,29]. These findings suggest that frontal theta oscillations may serve as a neural marker of cognitive control engagement, reflecting the brain’s attempt to optimize performance under challenging conditions. Functional connectivity studies further indicate that frontal-parietal theta coherence increases during decision-making, suggesting that theta-band synchronization facilitates communication between these regions [30,31]. Recent causal evidence using transcranial magnetic stimulation demonstrates that lateral prefrontal theta oscillations actively drive computational mechanisms for conflict expectation and cognitive resource allocation [32], suggesting theta plays a proactive role in cognitive control rather than merely reflecting processing difficulty.

The temporal dynamics of theta oscillations during decision-making reveal distinct phases of cognitive processing. Early theta responses, occurring within the first 200 ms after stimulus presentation, appear to reflect initial sensory processing and attention allocation [17]. Later theta activity, particularly in frontal regions, has been associated with evidence accumulation and decision formation processes [27]. This temporal organization suggests that theta oscillations provide a dynamic framework for coordinating different stages of perceptual decision-making, from initial sensory analysis to final response selection [13,26].

Decision confidence constitutes a fundamental metacognitive process in perceptual decision-making, representing the subjective evaluation of decision accuracy that influences subsequent behavioral adjustments and learning mechanisms [33]. Neuroimaging investigations have identified prefrontal cortical regions as critical substrates for confidence judgments and metacognitive monitoring. Selective manipulations of prefrontal activity using neurofeedback can alter confidence reports without affecting perceptual accuracy [34], while prefrontal lesions impair confidence calibration despite preserved decision performance [33], demonstrating dissociable neural substrates for decision-making and metacognition evidence indicates that theta-band oscillations may constitute a neurophysiological mechanism mediating the relationship between cognitive control processes and metacognitive evaluation. Specifically, prefrontal theta activity demonstrates significant associations with metacognitive adequacy, exhibiting positive correlations with the capacity to discriminate between accurate and inaccurate decisions [35]. Central frontal theta oscillations have been proposed to function as a neural broadcasting system that signals cognitive control demands based on internal assessments of decision quality [36]. These findings suggest that enhanced frontal theta activity associated with clear sensory evidence and efficient cognitive control engagement may reflect both successful task performance and elevated decision confidence.

Despite extensive research on theta oscillations in cognitive control, several key questions remain unresolved. While theta activity increases with cognitive demand in conflict tasks [27], its response to parametric variations in perceptual evidence quality-independent of response conflict-remains unclear. Most studies manipulate conflict or task difficulty rather than stimulus clarity, leaving uncertain whether theta reflects processing effort or evidence quality. Additionally, although evoked and induced oscillations are conceptually distinct [37] and methodological differences between ERP—based and single-trial analysis are well reorganized [38], few studies systematically compared these approaches within the same perceptual decision-making dataset. Such comparisons would clarify the sensitivity of each method and how they jointly inform interpretation. Furthermore, while frontal theta has been linked to metacognitive monitoring [35], its relationship to decision confidence during perceptual decision making remains poorly characterized.

The present study addresses these gaps by examining theta power and connectivity under parametric stimulus clarity manipulation (0%, 40%, 55% noise) using complementary analytical approaches (ERP—based single-trial, evoked/induced decomposition). We systematically apply established techniques to classify theta’s relationship to perceptual evidence quality and demonstrate how different analytical methods provide complementary insights into the same neural phenomena.

## 2. Materials and Methods

### 2.1. Dataset

EEG data were obtained from a previously published study [39] that investigated oscillatory network dynamics during face and house image perception. The original experimental procedures are summarized below, followed by a description of the analytical methods used for theta oscillation analysis in the current study.

### 2.2. Participants

The study included twenty-six neurologically healthy volunteers, comprising 21 males and five females, aged between 22 and 38 years, with a mean age of 26.3 years and a standard deviation of 4.7 years. Prior to participation, each volunteer provided written informed consent. The study protocol was approved by the Institutional Review Board of Georgia State University. Data from three participants were excluded from the final analyses due to either poor behavioral performance or excessive EEG artifacts and noise that could not be reliably corrected.

### 2.3. Stimuli

The experimental stimuli consisted of a total of 28 images, evenly divided between faces and houses (14 images per category). Face images were sourced from the Ekman series [39]. For each image, Fast Fourier Transforms (FFT) were computed, generating 28 magnitude matrices and 28 phase matrices. An average magnitude matrix was then calculated across all images. New images were created by performing an inverse FFT (IFFT) using this average magnitude matrix combined with the phase matrix of each individual image. The phase matrix used for the IFFT followed a linear arrangement to preserve the essential spatial structure of the original images.

### 2.4. Experimental Design

**Figure 1:**
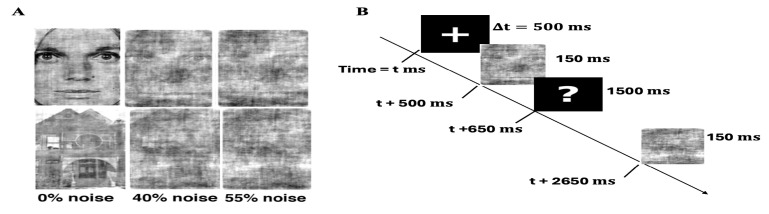
Task Paradigm (**A**) Visual stimuli used in the perceptual decision-making task. Examples of face (top row) and house (bottom row) stimuli across three noise levels: 0% noise (clear), 40% noise, and 55% noise. Phase noise was added using Fourier transform techniques while preserving identical power spectra across conditions. Participants categorized each image as face or house during 150 ms presentation windows. (**B**) Experimental procedure and trial structure. Each trial began with a fixation cross (+) for 500 ms, followed by stimulus presentation (face or house) for 150 ms, then a response prompt (?) for 1500 ms during which participants made face/house categorizations. Inter-trial interval was 1500 ms.

Before starting the experimental task, participants were given a brief explanation of the task paradigm. They were seated in a darkened room, with the only source of light coming from the experimenter’s computer screen and maintained a viewing distance of approximately 60 cm using a chin rest. A schematic of the experimental paradigm is shown in Figure 1. The experiment consisted of four blocks, each containing 168 trials, resulting in a total of 672 trials, with 224 trials for each noise level. On each trial, a small fixation cross (“+”) was displayed at the center of the screen for 500 ms. This was followed by the presentation of a stimulus for 150 ms, after which a black screen with a question mark (“?”) appeared for 1500 ms. During this interval, participants were allowed to indicate their decision by identifying the stimulus as either a face or a house by pressing the corresponding key on the keyboard.

### 2.5. Data Acquisition and Preprocessing

EEG signals were recorded using a 64-channel system from Brain Vision LLC (Morrisville, NC, USA, www.brainvision.com), with analog signals digitized at a sampling rate of 500 Hz. Electrode impedances were maintained below 10 kΩ throughout the recordings. Participants were instructed to minimize eye blinks, head movements, and swallowing during the experiment. The EEG data were digitally band-pass filtered between 1 and 100 Hz and notch filtered at 60 Hz to remove line noise. Ocular artifacts, such as blinks, were corrected using independent component analysis (ICA). Data from three participants were excluded from further analyses due to either excessive EEG artifacts and noise or poor behavioral performance. All preprocessing procedures were carried out using Brain Vision Analyzer 2.0 (www.brainproducts.com).

### 2.6. Software and Analysis Parameters

All analyses were conducted using MATLAB R2021b (The MathWorks, Natick, MA, USA) with custom scripts. Complex Morlet wavelet decomposition used custom implementations based on [40] with central frequency parameter k_0_ ≈ 4.73 and Fourier factor = 1.0, computed at 50 linearly spaced frequencies (1–50 Hz) with zero-padding to the next power of 2. Current source density transformation used CSD Toolbox v1.1 [41] with default parameters: m-constant = 4, lambda = 1 × 10^−5^, head radius = 10 cm. Epochs spanned −226 to 423 ms relative to stimulus onset (325 time points at 500 Hz sampling rate). Statistical comparisons used Wilcoxon signed-rank tests with FDR correction [42].

### 2.7. Data Analysis

#### 2.7.1. EEG Recording and Initial Preprocessing

EEG data were collected using a 64-channel Brain Vision system with a 500 Hz sampling rate. Electrode impedance was maintained below 10 kΩ throughout the recording session to ensure optimal signal quality. The data were bandpass filtered between 1 and 100 Hz during acquisition, with a 60 Hz notch filter applied to eliminate electrical line noise. Independent component analysis (ICA) was subsequently used to correct for ocular artifacts using Brain Vision Analyzer 2.0, providing systematic removal of eye movement and blink-related contamination from the neural signals.

#### 2.7.2. Epoching and High-Pass Filtering

Continuous EEG data were epoched into segments based on stimulus onset, with analysis focused exclusively on post-stimulus intervals to capture task-related neural responses. The analysis in this study included only the epochs for face-perception. Following initial epoching, a high-pass filtering procedure was implemented using a 10th-order FIR filter with a 3 Hz cutoff frequency and zero-phase distortion characteristics, implemented via the filtfilt.m function to ensure temporal precision. The cutoff frequency was normalized relative to the Nyquist frequency (fs/2), and filter coefficients were generated using the fir1 function with ‘high’ specification. This 3 Hz high-pass filter effectively eliminated slow-wave artifacts and low-frequency noise while preserving the integrity of theta-band oscillations (3–7 Hz) and higher-frequency neural signals.

#### 2.7.3. Time-Frequency Analysis

Time-frequency wavelet spectra were generated to visualize theta power distributions across electrodes and time points for each experimental condition. Theta oscillation analysis focused on the 3–7 Hz frequency band, consistent with recent literature defining theta activity within this range [43]. This definition captures the core theta frequency range while minimizing potential overlap with the theta-alpha transition zone and aligns with contemporary studies of theta oscillations in perceptual and decision-making contexts. The temporal analysis was restricted to post-stimulus intervals to capture task-specific neural responses without contamination from pre-stimulus baseline activity. The temporal window of interest extended from stimulus onset through 400 ms post-stimulus (0–400 ms), encompassing the early perceptual processing and decision formation period identified through systematic examination of grand average event-related potential patterns across participants.

Time-frequency decomposition was performed using complex Morlet wavelet transform. The complex Morlet wavelet analysis employed a central frequency parameter (ω_0_) and computed spectral matrices across trials for each electrode pair. Auto- and cross-spectra were calculated by taking the wavelet transform of individual trials and computing the complex conjugate products, with final spectral estimates obtained by averaging across trials [38]. Two complementary analytical approaches were implemented to provide methodological validation and comprehensive characterization of theta dynamics.

The first method involved ERP-based analysis, wherein event-related potentials were computed by averaging EEG signals across trials for each participant and experimental condition. Current source density transformation was subsequently applied to these averaged ERPs using a spherical spline algorithm, and theta band power was extracted from the resulting CSD-transformed data.

The second approach employed single-trial analysis, where CSD transformation was applied directly to individual trials prior to any averaging procedures, with theta power values extracted and subsequently averaged across trials for each participant to yield robust estimates of oscillatory activity.

#### 2.7.4. Clarity Level Specification and Pooling

The validity of this pooling approach was formally tested through supplementary three level analyses (0%, 40%, 55%) examining frontal and parietal theta power separately for each clarity level (Supplementary Figure S1, Supplementary Table S1). Statistical comparisons used Wilcoxon signed-rank tests with FDR correction (Benjamini–Hochberg procedure, α = 0.05) across all six pairwise comparisons (3 frontal + 3 parietal). These analyses confirmed that frontal theta power exhibited a step-function pattern, with clear stimuli producing significantly higher power than both noise levels (both p_FDR < 0.01), while the two noise levels did not differ from each other (p_FDR = 0.35). Three-level analysis of frontoparietal imaginary coherence revealed a graded pattern, with high noise (M = 0.20) differing significantly from both clear (M = −0.19; Z = −3.39, p_FDR = 0.001) and moderate levels (M = −0.11; Z = −3.57, p_FDR = 0.001), while clear and moderate did not differ (Z = −1.13, p_FDR = 0.26) (Supplementary Figure S2, Supplementary Table S2). The moderate condition thus represents an intermediate state for connectivity, showing frontal-leading coordination like clear stimuli but distinguishable from the parietal-leading pattern at high noise. This pattern validates pooling while acknowledging measure-specific differences, with power showing a threshold effect and connectivity showing a graded shift in phase relationships.

#### 2.7.5. Current Source Density (CSD) Transformation

Current source density transformation was implemented using the established CSD Toolbox [41], employing spherical spline interpolation with default parameters: m-constant (spline flexibility) = 4, lambda (smoothing parameter) = 1 × 10^−5^, and head radius = 10 cm. Electrode locations from the 64-channel standard 10–20 system configuration were used for the spherical spline interpolation. This transformation served the critical function of eliminating volume conduction effects and reference electrode artifacts, thereby enhancing spatial resolution and ensuring that observed neural activity patterns reflected genuine local current sources rather than spurious electrical conduction through brain tissue and scalp.

#### 2.7.6. Functional Connectivity Analysis

Imaginary coherence analysis was conducted between electrode pairs representing frontal (F1, Fz, F2) and parietal (P1, Pz, P2) regions to characterize inter-regional neural communication patterns during post-stimulus processing. The coherence and imaginary coherence calculations were performed using the spectral matrices obtained from the complex Morlet wavelet transform.

Coherence was computed as: $Coh=\frac{\left|S\left(i,j\right)\right|}{\sqrt{S\left(i,i\right)*S\left(j,j\right)}}$, where *S*(*i*,*j*) represents the cross-spectral density between electrode pairs *i* and *j*, and *S*(*i*,*i*), S(*j*,*j*) represent the respective auto-spectral densities. Imaginary coherence was calculated as: $iCoh=im\left(\frac{S\left(i,j\right)}{\sqrt{S\left(i,i\right)*S\left(j,j\right)}}\right)\;$which is mathematically equivalent to taking the imaginary component of the normalized cross-spectrum. Since the denominator √(*S*(*i*,*i*) × *S*(*j*,*j*)) is real and positive, this operation extracts the imaginary component of the complex cross-spectrum normalized by the geometric mean of the auto-spectra.

Imaginary coherence values are signed, with the sign indicating the direction of the phase relationship between regions: negative values indicate that frontal activity leads parietal activity in phase (frontal precedes parietal), while positive values indicate parietal-leading phase relationships. The magnitude of iCoh reflects the strength of phase-lagged coupling. This metric specifically measures phase-lagged connectivity while excluding zero-lag interactions that may arise from volume conduction.

Analysis was restricted to post-stimulus intervals within the 3–7 Hz theta frequency range to maintain consistency with power analysis parameters and focus on task-relevant neural dynamics. The combination of CSD transformation and imaginary coherence analysis provided a dual artifact removal approach, with CSD transformation eliminating volume conduction effects and imaginary coherence specifically excluding zero-lag interactions that may arise from spurious electrical conduction. This conservative analytical framework ensured that observed connectivity patterns reflected genuine phase-lagged neural communication between brain regions during task performance.

#### 2.7.7. Connectivity Decomposition: Phase-Locked vs. Induced Components

To isolate the neural dynamics driving functional coupling, we decomposed connectivity into phase-locked and non-phase-locked components using Imaginary Coherence (iCoh). This metric was selected to mitigate the influence of volume conduction by focusing on the imaginary part of the cross-spectrum. Connectivity was assessed over the 0–400 ms post-stimulus window. The first component analyzed was total connectivity, estimated by calculating iCoh on a trial-by-trial basis and subsequently averaging the results. This measure represents the aggregate functional coupling, encompassing both phase-locked (evoked) and non-phase-locked (induced) oscillatory synchronization.

Conversely, the evoked component was isolated to strictly represent coupling locked to stimulus onset. This was achieved by computing iCoh directly from the trial-averaged Event-Related Potentials (ERPs), reflecting only phase-consistent interactions that maintain a stable phase relationship across trials.

Furthermore, the non-phase-locked, or induced component was isolated using a specific residual-based subtraction method. First, the condition-specific ERP was subtracted from each individual trial in the time domain. iCoh was then calculated for each resulting residual trial and averaged. By removing the common evoked component, this approach ensures that the resulting induced connectivity reflects genuine trial-to-trial coupling independent of stimulus-locked phase reset, thereby avoiding spurious correlations driven by the evoked response.

#### 2.7.8. Decomposition of Oscillatory Activity 

To dissociate neural oscillatory activity into phase-locked (evoked) and non-phase-locked (induced) components, we employed standard time-frequency decomposition procedures [37]. This decomposition distinguishes stimulus-locked activity that maintains consistent phase relationships across trials from ongoing oscillations whose phase varies trial-to-trial.

Total Power (Average-of-Power): Time-frequency decomposition was applied to each individual trial using wavelet convolution. Power values (squared magnitude of complex wavelet coefficients) were computed for each trial and subsequently averaged across all trials. This “average-of-power” approach captures aggregate oscillatory activity encompassing both evoked and induced components.

Evoked Power (Power-of-Average): Individual trials were first averaged in the time domain to generate the event-related potential (ERP), eliminating non-phase-locked activity through destructive interference. Time-frequency decomposition was then applied exclusively to this trial-averaged waveform. This “power-of-average” approach reflects only phase-locked activity maintaining consistent phase relationships across trials.

Induced Power (Subtraction Method): Induced power was calculated by subtracting evoked power from total power in the linear power domain before logarithmic transformation, preserving the mathematical relationship where total power equals the sum of evoked and induced power. Final values were expressed in decibels: Induced Power (dB) = 10 × log_10_(Total Power − Evoked Power).

Methodological Considerations: This decomposition approach has well-established limitations [37,38]. First, the evoked/induced distinction represents a methodological separation rather than necessarily reflecting distinct neural generators. Second, subtraction-based induced power estimates can be sensitive to trial-to-trial variability in evoked response latency or amplitude, potentially redistributing evoked variance into the induced estimate. Third, the decomposition assumes linear additivity in the power domain, which may not fully capture nonlinear interactions between phase-locked and ongoing oscillations. Despite these limitations, this approach provides a principled framework for distinguishing stimulus-locked from ongoing oscillatory dynamics and has been widely validated in cognitive neuroscience research.

#### 2.7.9. Statistical Analysis

Statistical comparisons between clear and noisy stimulus conditions employed non-parametric Wilcoxon signed-rank tests appropriate for our paired within-subject experimental design. Effect sizes were quantified using Cohen’s dz, calculated as Z/√*n* for standardized effect size estimation from Wilcoxon tests.

To control for multiple comparisons across related tests within each analysis domain, we applied False Discovery Rate (FDR) correction using the Benjamini–Hochberg procedure (α = 0.05). Correction families were clearly defined for each analysis domain: (1) Power analyses: FDR applied across 4 tests (2 regions [frontal, parietal] × 2 methods [ERP-based, single-trial]); (2) Connectivity analyses: FDR applied across 2 tests (ERP-based vs. single-trial imaginary coherence); (3) Connectivity decomposition: FDR applied across 2 tests (evoked vs. induced components); (4) Three-level analyses: FDR applied across 6 tests (3 frontal + 3 parietal pairwise comparisons).

The FDR procedure controls for multiple testing across these related contrasts within each analysis family. Wilcoxon signed-rank tests provided the test statistics for individual comparisons, while FDR correction addressed the multiple comparisons problem. No corrections were applied across time points, frequencies, or individual electrodes, as analyses focused on pre-defined ROIs (frontal: F1, Fz, F2; parietal: P1, Pz, P2) and a single frequency band (3–7 Hz theta). Statistical significance was determined using FDR-corrected *p*-values (q-values) with a threshold of q < 0.05.

Time Window Selection: The 0–400 ms post-stimulus window was selected based on visual inspection of grand-average event-related potential patterns and time-frequency plots, capturing the early perceptual processing and decision formation period. This data-guided selection was validated through robustness checks: a narrower window (100–300 ms) yielded consistent frontal theta power effects (*p* < 0.001), demonstrating that results were not dependent on specific window boundaries.

## 3. Results

**Figure 2:**
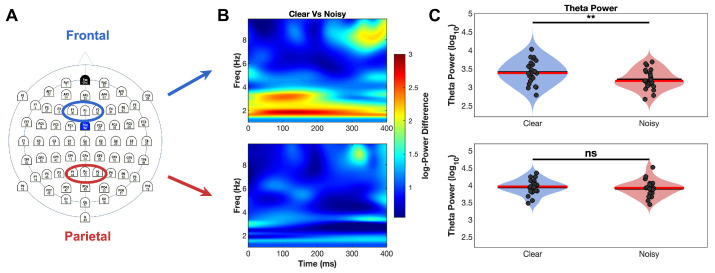
Average theta power analysis for clear and noisy face stimulus conditions. (**A**) 64-channel electrode layout highlighting frontal ROI and parietal ROI used for analysis. (**B**) Time-frequency plots comparing clear vs. noisy stimuli for frontal (top) and parietal (bottom) regions. Color scale represents power differences, with warm colors indicating higher power for clear stimuli. Clear stimuli show sustained theta enhancement (3–7 Hz) from 0 to 400 ms post-stimulus, particularly in frontal regions. (**C**) Theta power analysis (0–400 ms window) demonstrating significant modulation in frontal regions (top, corrected *p* < 0.001,** = significant) but not parietal regions (bottom, corrected *p* = 0.93, ns = not significant), confirming the frontal specificity of stimulus clarity effects. Blue bars = clear stimuli, red bars = noisy stimuli.

**Figure 3:**
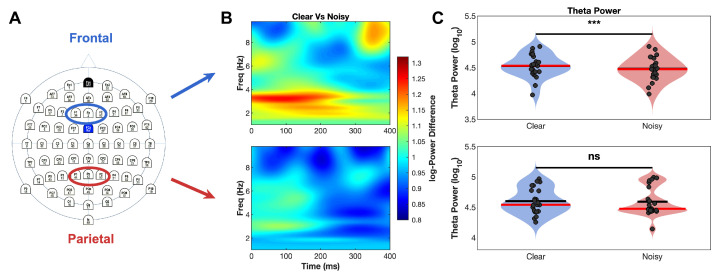
Single-trial theta power analysis for clear and noisy face stimulus conditions. (**A**) 64-channel electrode layout highlighting frontal ROI and parietal ROI used for analysis. (**B**) Time-frequency plots comparing clear vs. noisy stimuli for frontal (top) and parietal (bottom) regions from single-trial CSD analysis. Color scale represents power differences, with warm colors indicating higher power for clear stimuli. Effects are more subtle than ERP-based analysis but show similar spatial and temporal patterns. (**C**) Power spectrum analysis (0–400 ms window) showing significant frontal modulation (top, corrected *p* < 0.001, ***) but not parietal modulation (bottom, corrected *p* = 0.99, ns = not significant), consistent with the frontal-specific pattern observed in ERP-based analysis. Blue bars = clear stimuli, red bars = noisy stimuli.

**Figure 4:**
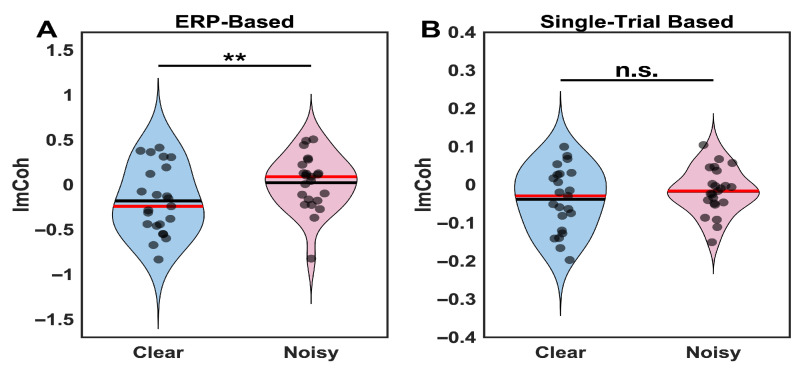
Frontoparietal theta connectivity: ERP-based vs. single-trial analysis of clear and noisy face stimulus conditions. (**A**) ERP-based (phase-locked) imaginary coherence showing significant differences between clear and noisy conditions (corrected *p* = 0.009, dz = −0.61). Clear stimuli exhibit more negative imaginary coherence values, indicating enhanced frontal-leading phase-lagged coupling. (**B**) Single-trial (total) imaginary coherence showing no significant differences between conditions (corrected *p* = 0.118, dz = −0.37). The dissociation between methods reflects differential sensitivity to phase-locked versus ongoing oscillatory components. Violin plots show distribution of individual participant values (dots), with red horizontal lines indicating median values. ** *p* < 0.01, n.s. = not significant.

**Figure 5:**
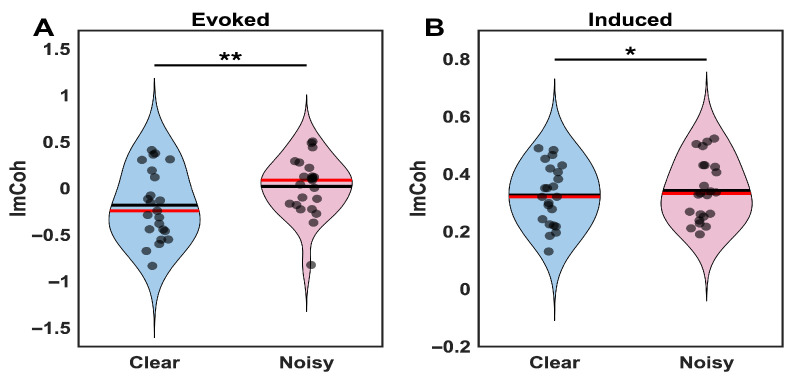
Frontoparietal theta connectivity decomposition for clear and noisy face image conditions. (**A**) Evoked (phase-locked) imaginary coherence was significantly more negative for clear than noisy stimuli (corrected *p* = 0.009, dz = −0.61). (**B**) Induced (non-phase-locked) imaginary coherence was significantly lower for clear than noisy stimuli (corrected *p* = 0.049, dz = −0.36), with opposite directionality and smaller magnitude. Violin plots show distributions (blue = clear, pink = noisy); red lines = medians. ** *p* < 0.01; * *p* < 0.05.

All primary analyses compared clear (0% noise) versus noisy (pooled 40% 55% noise) face image conditions (Figure 2, Figure 3, Figure 4 and Figure 5). This binary contrast was validated through supplementary three-level analyses. Frontal theta power exhibited a step-function pattern, with both noise levels eliciting similar responses that differed significantly from clear stimuli (Supplementary Figure S1, Supplementary Table S1). Frontoparietal imaginary coherence showed a graded pattern, with moderate noise serving as an intermediate state between clear and high noise (Supplementary Figure S2, Supplementary Table S2). Parietal regions showed no consistent clarity modulation in theta power, confirming the frontal specificity of power effects reported below. All analyses were conducted on 23 participants after excluding 3 due to excessive artifacts or poor behavioral performance (see Section 2.2). Trial counts after artifact rejection averaged (mean ± SD across participants): Clear condition: 96.0 ± 8.4 trials; Moderate noise: 93.2 ± 9.6 trials; High noise: 53.2 ± 20.7 trials; Pooled noisy condition: 146.3 ± 27.6 trials. The lower trial count for high noise reflects greater artifact susceptibility in this more challenging condition.

### 3.1. Theta Power Analysis

#### 3.1.1. Method Comparison: ERP-Based vs. Single-Trial Analysis

Both analytical approaches revealed significant theta modulation by stimulus clarity, though they differed in sensitivity and effect magnitude. The ERP-based analysis demonstrated robust frontal theta increases for clear versus noisy stimuli during the 100–300 ms post-stimulus window (Figure 2 for the CSD time series of the ERPs and Figure 3 for the CSD time series of the single-trials). This effect was large and statistically significant (*p* < 0.001), with clear stimuli showing higher theta power than noisy stimuli. Parietal regions showed no significant differences between conditions (*p* = 0.62), confirming the frontal specificity of theta modulation.

The single-trial analysis yielded more conservative but consistent findings. Frontal regions again exhibited significant theta modulation (*p* < 0.001), though with smaller effect sizes reflecting the conservative nature of this approach. Parietal regions remained non-significant (*p* = 0.99), maintaining the frontal specificity pattern observed in the ERP-based analysis. These results validate theta modulation effects across both analytical methods while demonstrating that ERP-based approaches provide greater sensitivity for detecting stimulus-related neural changes.

#### 3.1.2. Temporal Dynamics

Theta power differences evolved systematically throughout the post-stimulus period. Differences emerged rapidly within 100 ms of stimulus presentation, peaked between 150 and 250 ms, and sustained throughout the entire 400 ms analysis window. This temporal progression aligns with established stages of perceptual processing and cognitive control engagement, suggesting that theta oscillations reflect dynamic attentional resource allocation during decision-making under varying stimulus clarity.

### 3.2. Frontal-Parietal Connectivity Analysis

The imaginary coherence analysis revealed an estimator-dependent dissociation between ERP-based and single-trial approaches. ERP-based imaginary coherence analysis demonstrated significant differences between clear and noisy conditions (*p* = 0.009), with clear stimuli exhibiting negative imaginary coherence values while noisy stimuli showed values near zero. This pattern indicates that stimulus clarity modulates frontoparietal neural synchronization during perceptual decision-making.

In contrast, single-trial imaginary coherence analysis showed no significant differences between clear and noisy conditions (*p* > 0.05). This dissociation between analytical methods suggested different sensitivity to connectivity components rather than necessarily distinct neural mechanisms. To clarify the nature of this modulation, we decompose connectivity into evoked (phase-locked) and ongoing (non-phase-locked) components (Section 3.2.1).

#### 3.2.1. Connectivity Decomposition: Evoked vs. Induced Components

To clarify the nature of the connectivity modulation and determine whether frontoparietal effects were confined to evoked components, we decomposed frontoparietal imaginary coherence into evoked (phase-locked) and induced (non-phase-locked) components (Figure 5). Evoked connectivity, computed from trial-averaged ERPs, showed significant differences between clear and noisy conditions (Z = −2.86, corrected *p* = 0.009, dz = −0.61). Clear stimuli exhibited more negative imaginary coherence values (M = −0.187, SD = 0.371) compared to noisy stimuli (M = 0.042, SD = 0.308), indicating enhanced phase-lagged coupling when perceptual evidence was reliable. Induced connectivity, computed from single-trial residuals after ERP subtraction, also showed significant differences between conditions (Z = −1.97, corrected *p* = 0.049, dz = −0.36), but importantly, with opposite directionality: clear stimuli showed slightly lower induced coupling values (M = 0.327, SD = 0.100) than noisy stimuli (M = 0.341, SD = 0.101). The effect size for evoked connectivity was 1.7-fold larger than for induced connectivity. These results indicate that the dissociation between ERP-based and single-trial connectivity analyses primarily reflects differences in sensitivity to evoked versus induced components, rather than distinct communication mechanisms. Stimulus clarity modulates frontoparietal theta coupling through both phase-locked and ongoing oscillatory processes, though with different magnitudes and opposing directions. The evoked, phase-locked component dominates the overall effect, explaining why ERP-based methods detected robust connectivity differences: they are highly sensitive to stimulus-locked synchronization. Single-trial methods yielded null results because the opposing induced component partially canceled the larger evoked effect when both components contributed to the combined estimate. This pattern underscores that the connectivity evidence is estimator-dependent and appears mainly evoked/phase-locked under the current analytical choices, with a secondary opposing contribution from non-phase-locked activity.

### 3.3. Induced Oscillatory Activity

#### Induced Theta Power

Induced power in the theta band did not significantly differ between Clear and Noisy conditions in either the Frontal (*p* = 0.0625) or Parietal (*p* = 1.0000) regions. Although the Frontal region showed a slight numerical increase in power for the Clear condition, this difference did not reach statistical significance. The results suggest that the clarity of the stimulus did not substantially modulate induced oscillatory power in these regions.

## 4. Discussion

Our results demonstrate that theta oscillations, particularly in frontal regions, play a critical role in visual perceptual decision-making, with power modulated by stimulus clarity. The finding that clear stimuli evoked higher frontal theta power than noisy stimuli was initially counterintuitive, as one might expect greater cognitive effort for ambiguous decisions. However, this pattern suggests that theta activity may reflect effective cognitive engagement and confident decision-making, rather than increased effort due to ambiguity. This interpretation aligns with recent theoretical frameworks proposing that frontal theta reflects the successful implementation of cognitive control rather than simply increased effort [27,44]. Clear stimuli may enable more efficient engagement of decision-making mechanisms, resulting in enhanced theta power that reflects optimal cognitive processing [45].

While our findings are consistent with the hypothesis that frontal theta reflects decision confidence—given that clear stimuli yielded both higher theta power and presumably easier, more confident decisions—we cannot directly test this relationship without explicit confidence ratings or trial-by-trial accuracy data. The original dataset did not record confidence judgments or preserve trial-level behavioral outcomes (accuracy, reaction time) in a format accessible for reanalysis. Therefore, the confidence interpretation remains hypothesis-level, supported by convergent evidence from prior studies linking prefrontal theta to metacognitive monitoring [33] and confidence reports [32], but requiring direct behavioral validation in future work. The most defensible interpretation of our results is that frontal theta power tracks stimulus clarity during early perceptual processing, with the functional relationship to decision confidence remaining an empirically testable prediction rather than a demonstrated finding.

A key finding of this study is the estimator-dependent pattern of frontoparietal connectivity modulation. The significant imaginary coherence differences observed in ERP-based analysis, but not in single-trial analysis, initially suggested that stimulus clarity specifically modulates stimulus-locked neural coordination while leaving intrinsic network communication intact. However, the connectivity decomposition (Section 3.2.1) revealed a more nuanced pattern: both evoked (phase-locked) and induced (non-phase-locked) components showed significant modulation by stimulus clarity, but with opposite directionality and different magnitudes. The evoked component exhibited stronger coupling for clear stimuli (more negative imaginary coherence values) with a large effect size (dz = −0.61), while the induced component showed slightly stronger coupling for noisy stimuli (dz = −0.36). Critically, the evoked effect was 1.7-fold larger, explaining why ERP-based methods detected robust connectivity differences while single-trial methods did not: the opposing induced component partially canceled the dominant evoked effect in combined estimates.

This pattern indicates that stimulus clarity modulates frontoparietal theta coupling through both phase-locked and ongoing oscillatory synchronization, though stimulus-locked coordination dominates. Rather than reflecting wholly distinct communication mechanisms, these findings demonstrate that different analytical approaches capture different aspects of the same neural dynamics: ERP-based methods are highly sensitive to robust, phase-locked coordination, while component-specific decomposition reveals the underlying complexity that standard single-trial approaches cannot fully capture.

The opposing directionality of evoked versus induced components warrants further investigation. One possibility is that clear stimuli evoke strong phase-locked frontoparietal synchronization for efficient evidence integration, while noisy stimuli may engage compensatory ongoing coupling that is not time-locked to stimulus onset. However, this interpretation must be tempered by acknowledging that these are estimator-dependent findings sensitive to methodological choices in connectivity computation. While Thatcher [46] warned about artifactually inflated coherence from signal averaging, our approach using CSD transformation and imaginary coherence combined with explicit evoked/induced decomposition reveals genuine stimulus-locked synchronization while acknowledging estimator-dependent sensitivity.

Both ERP-based and single-trial analyses demonstrated significant frontal theta power increases for clear versus noisy stimuli, with effects emerging within 100 ms and sustaining throughout the 400 ms analysis window. Frontal regions consistently dominated theta-band responses across both analytical methods, while parietal regions showed minimal modulation by stimulus clarity, suggesting that frontal areas implement primary cognitive control mechanisms during perceptual decision-making under uncertainty. Recent studies have demonstrated that frontal theta power during task preparation reflects cognitive effort allocation [47] and that theta dynamics track the developmental trajectory of cognitive control from childhood to adulthood [48], further supporting theta’s role as a marker of control engagement rather than mere difficulty.

This study illustrates the importance of decomposing connectivity into constituent components when interpreting EEG-based functional coupling. While our dual artifact removal approach combining CSD transformation with imaginary coherence provides a conservative measure of genuine inter-regional communication, the connectivity effects are primarily driven by phase-locked (evoked) activity, with a secondary and opposing contribution from non-phase-locked (induced) activity. This decomposition clarifies that ERP-based and single-trial methods capture different aspects of the same neural dynamics rather than entirely separate mechanisms, with methodological choices determining which components are most readily detected [48,49]. These findings collectively demonstrate that theta oscillations serve as dynamic markers of cognitive control engagement, with frontal regions playing a dominant role in adapting neural responses to varying perceptual demands.

Several methodological limitations should be acknowledged. Although EEG was recorded with a 64-channel montage providing relatively dense scalp coverage, analyses were performed in sensor space using CSD, limiting anatomical inference. Statistical testing was confined to a small a priori frontal–parietal ROI set as by Rajan and colleagues [30] and used electrode-wise FDR correction, which does not account for spatial correlations. Future studies should adopt full-scalp cluster-based permutation testing and volumetric source reconstruction methods (e.g., minimum norm, eLORETA, or beamforming). Nevertheless, despite these limitations, the present results provide converging evidence for a functional involvement of theta oscillations and theta-band connectivity in perceptual decision-making.

## 5. Conclusions

The present study demonstrates that frontal theta oscillations play a central role in perceptual decision-making, with both power and connectivity patterns selectively modulated by stimulus clarity. Clear stimuli elicited stronger frontal theta responses than noisy stimuli, a finding that emerged rapidly within 100 ms and persisted throughout the decision-making window. Rather than reflecting increased effort under ambiguity, frontal theta activity here appears to index effective cognitive engagement and confident decision-making when perceptual evidence is reliable. This interpretation is consistent with frameworks positioning theta as a neural marker of adaptive control, bridging sensory input with higher-order executive processes. While we did not directly measure decision confidence, the observed pattern—stronger theta for clear stimuli—is consistent with hypothesized links between frontal theta and confidence-related processing, though behavioral correlates would be needed to confirm this relationship.

Equally important, our connectivity decomposition revealed that stimulus clarity modulates frontoparietal theta coupling through both phase-locked and ongoing oscillatory mechanisms, though with different magnitudes and opposing directions. The dominant phase-locked component (1.7-fold larger effect size) indicates that clear stimuli primarily enhance stimulus-locked temporal coordination between frontal and parietal regions, consistent with models proposing that phase synchronization provides a temporal framework for inter-regional communication. The presence of a significant but opposing induced effect—with slightly stronger coupling for noisy stimuli—suggests potential compensatory mechanisms during ambiguous perception, though this secondary pattern requires further investigation.

This decomposition clarifies that the initial dissociation between ERP-based and single-trial analyses reflected methodological differences in sensitivity to phase-locked versus ongoing components rather than entirely distinct communication mechanisms. The opposing directions of evoked and induced effects explain why the combined single-trial analysis yielded null results: these components partially canceled each other despite both showing real modulation. This finding underscores the importance of decomposing connectivity into constituent components when interpreting EEG-based functional coupling.

Methodologically, this study illustrates the value of combining complementary analytical approaches with conservative connectivity measures. Current source density transformation eliminated volume conduction artifacts, while imaginary coherence specifically captured phase-lagged interactions. The decomposition into evoked and induced components revealed that different analytical methods are differentially sensitive to phase-locked versus ongoing synchronization, each providing valid but distinct perspectives on neural communication. ERP-based analyses proved highly sensitive to robust, stimulus-locked coordination, while component-specific decomposition revealed the underlying mechanisms that standard single-trial approaches cannot fully capture.

Taken together, these results provide new insight into how frontal theta oscillations support perceptual decision-making through complementary phase-locked and ongoing components: dominant stimulus-locked coordination that enhances with stimulus clarity and secondary ongoing coupling that may reflect compensatory processing during ambiguity. By demonstrating the distinct but complementary contributions through connectivity decomposition, the study emphasizes both the flexibility of stimulus-driven neural responses and the complexity of interpreting connectivity patterns across different analytical frameworks. These findings underscore that different analytical methods capture different aspects of the same underlying neural dynamics rather than entirely separate mechanisms. Future work incorporating explicit confidence ratings and behavioral correlates of theta activity would further clarify the functional significance of these oscillatory mechanisms in perceptual decision-making.

## Data Availability

The code and data used in this study have been made publicly available via the Open Science Framework (OSF) repository (https://osf.io/jsry2, accessed on 20 January 2026).

## Supplementary Materials

The following supporting information can be downloaded at: https://www.mdpi.com/article/10.3390/brainsci16020123/s1.

## References

[1] Green N., Heekeren H.R. (2009). Perceptual decision making: A bidirectional link between mind and motion. Prog. Brain Res..

[2] Maksimenko V.A., Kuc A., Frolov N.S., Khramova M.V., Pisarchik A.N., Hramov A.E. (2020). Dissociating Cognitive Processes During Ambiguous Information Processing in Perceptual Decision-Making. Front. Behav. Neurosci..

[3] Kelly S.P., O’Connell R.G. (2013). Internal and external influences on the rate of sensory evidence accumulation in the human brain. J. Neurosci..

[4] Stone C., Mattingley J.B., Bode S., Rangelov D. (2024). Distinct neural markers of evidence accumulation index metacognitive processing before and after simple visual decisions. Cereb. Cortex.

[5] Heekeren H.R., Marrett S., Ungerleider L.G. (2008). The neural systems that mediate human perceptual decision making. Nat. Rev. Neurosci..

[6] Summerfield C., de Lange F.P. (2014). Expectation in perceptual decision making: Neural and computational mechanisms. Nat. Rev. Neurosci..

[7] Ratcliff R., McKoon G. (2008). The diffusion decision model: Theory and data for two-choice decision tasks. Neural Comput..

[8] Shin S., Oh J., Kim S.K., Lee Y.-S., Kim S.J. (2025). Quantitative dynamics of neural uncertainty in sensory processing and decision-making during discriminative learning. Exp. Mol. Med..

[9] Gold J.I., Shadlen M.N. (2007). The neural basis of decision making. Annu. Rev. Neurosci..

[10] Okazawa G., Kiani R. (2023). Neural Mechanisms That Make Perceptual Decisions Flexible. Annu. Rev. Physiol..

[11] Basar E. (2013). Brain oscillations in neuropsychiatric disease. Dialogues Clin. Neurosci..

[12] van Bree S., Levenstein D., Krause M.R., Voytek B., Gao R. (2025). Processes and measurements: A framework for understanding neural oscillations in field potentials. Trends Cogn. Sci..

[13] Fries P. (2015). Rhythms for Cognition: Communication through Coherence. Neuron.

[14] Luo Y., Meng X., Zhou G., Zhou J., Luo Y.-J., Ai H., Zelano C., Chen F., Xu P. (2024). Oscillatory mechanisms of intrinsic human brain networks. NeuroImage.

[15] Kahana M.J. (2006). The cognitive correlates of human brain oscillations. J. Neurosci..

[16] Klimesch W. (1999). EEG alpha and theta oscillations reflect cognitive and memory performance: A review and analysis. Brain Res. Brain Res. Rev..

[17] Clayton M.S., Yeung N., Cohen Kadosh R. (2015). The roles of cortical oscillations in sustained attention. Trends Cogn. Sci..

[18] Guth T.A., Brandt A., Reinacher P.C., Schulze-Bonhage A., Jacobs J., Kunz L. (2025). Theta-phase locking of single neurons during human spatial memory. Nat. Commun..

[19] Sauseng P., Klimesch W. (2008). What does phase information of oscillatory brain activity tell us about cognitive processes?. Neurosci. Biobehav. Rev..

[20] Navi F.F.T., Heysieattalab S., Raoufy M.R., Sabaghypour S., Nazari M., Nazari M.A. (2024). Adaptive closed-loop modulation of cortical theta oscillations: Insights into the neural dynamics of navigational decision-making. Brain Stimul..

[21] Engel A.K., Fries P., Singer W. (2001). Dynamic predictions: Oscillations and synchrony in top-down processing. Nat. Rev. Neurosci..

[22] Martinovic J., Busch N.A. (2011). High frequency oscillations as a correlate of visual perception. Int. J. Psychophysiol..

[23] Sauseng P., Griesmayr B., Freunberger R., Klimesch W. (2010). Control mechanisms in working memory: A possible function of EEG theta oscillations. Neurosci. Biobehav. Rev..

[24] Herweg N.A., Solomon E.A., Kahana M.J. (2020). Theta Oscillations in Human Memory. Trends Cogn. Sci..

[25] Kapetaniou G.E., Vural G., Soutschek A. (2025). Frontoparietal theta stimulation causally links working memory with impulsive decision making. Cortex.

[26] Lisman J.E., Jensen O. (2013). The theta-gamma neural code. Neuron.

[27] Cavanagh J.F., Frank M.J. (2014). Frontal theta as a mechanism for cognitive control. Trends Cogn. Sci..

[28] Cavanagh J.F., Zambrano-Vazquez L., Allen J.J. (2012). Theta lingua franca: A common mid-frontal substrate for action monitoring processes. Psychophysiology.

[29] Nigbur R., Ivanova G., Sturmer B. (2011). Theta power as a marker for cognitive interference. Clin. Neurophysiol..

[30] Rajan A., Siegel S.N., Liu Y., Bengson J., Mangun G.R., Ding M. (2019). Theta Oscillations Index Frontal Decision-Making and Mediate Reciprocal Frontal-Parietal Interactions in Willed Attention. Cereb. Cortex.

[31] Siegel M., Donner T.H., Engel A.K. (2012). Spectral fingerprints of large-scale neuronal interactions. Nat. Rev. Neurosci..

[32] Martinez-Molina M.P., Valdebenito-Oyarzo G., Soto-Icaza P., Zamorano F., Figueroa-Vargas A., Carvajal-Paredes P., Stecher X., Salinas C., Valero-Cabre A., Polania R. (2024). Lateral prefrontal theta oscillations causally drive a computational mechanism underlying conflict expectation and adaptation. Nat. Commun..

[33] Fleming S.M., Dolan R.J. (2012). The neural basis of metacognitive ability. Philos. Trans. R. Soc. Lond. B Biol. Sci..

[34] Cortese A., Amano K., Koizumi A., Kawato M., Lau H. (2016). Multivoxel neurofeedback selectively modulates confidence without changing perceptual performance. Nat. Commun..

[35] Wokke M.E., Cleeremans A., Ridderinkhof K.R. (2017). Sure I’m Sure: Prefrontal Oscillations Support Metacognitive Monitoring of Decision Making. J. Neurosci..

[36] Wokke M.E., Achoui D., Cleeremans A. (2020). Action information contributes to metacognitive decision-making. Sci. Rep..

[37] Tallon-Baudry C., Bertrand O. (1999). Oscillatory gamma activity in humans and its role in object representation. Trends Cogn. Sci..

[38] Cohen M.X. (2014). Analyzing Neural Time Series Data: Theory and Practice.

[39] Chand G.B., Lamichhane B., Dhamala M. (2016). Face or House Image Perception: Beta and Gamma Bands of Oscillations in Brain Networks Carry Out Decision-Making. Brain Connect..

[40] Torrence C., Compo G.P. (1998). A Practical Guide to Wavelet Analysis. Bull. Am. Meteorol. Soc..

[41] Kayser J., Tenke C.E. (2006). Principal components analysis of Laplacian waveforms as a generic method for identifying ERP generator patterns: II. Adequacy of low-density estimates. Clin. Neurophysiol..

[42] Benjamini Y., Hochberg Y. (1995). Controlling the False Discovery Rate: A Practical and Powerful Approach to Multiple Testing. J. R. Stat. Soc. Ser. B (Methodol.).

[43] Ostrowski J., Rose M. (2024). Increases in pre-stimulus theta and alpha oscillations precede successful encoding of crossmodal associations. Sci. Rep..

[44] Cooper P.S., Wong A.S., Fulham W.R., Thienel R., Mansfield E., Michie P.T., Karayanidis F. (2015). Theta frontoparietal connectivity associated with proactive and reactive cognitive control processes. Neuroimage.

[45] Hajihosseini A., Holroyd C.B. (2013). Frontal midline theta and N200 amplitude reflect complementary information about expectancy and outcome evaluation. Psychophysiology.

[46] Thatcher R.W. (2012). Coherence, phase differences, phase shift, and phase lock in EEG/ERP analyses. Dev. Neuropsychol..

[47] Arnau S., Liegel N., Wascher E. (2024). Frontal midline theta power during the cue-target-interval reflects increased cognitive effort in rewarded task-switching. Cortex.

[48] Morales S., Buzzell G.A. (2025). EEG time-frequency dynamics of early cognitive control development. Dev. Cogn. Neurosci..

[49] Bastos A.M., Schoffelen J.M. (2015). A Tutorial Review of Functional Connectivity Analysis Methods and Their Interpretational Pitfalls. Front. Syst. Neurosci..

