# Supplementary material for: Frontal Theta Oscillations in Perceptual Decision-Making Reflect Cognitive Control and Confidence
Source: Brain Sci. 2026 Jan 23;16(2):123. doi: 10.3390/brainsci16020123 (PMC12937794; doi:10.3390/brainsci16020123)
Supplement: Supplementary file 1 [file brainsci-16-00123-s001.zip › brainsci-4091956-supplementary-1.pdf]

SUPPLEMENTARY MATERIALS

Frontal Theta Oscillations in Perceptual Decision-Making Reflect Cognitive Control and Confidence

Rashmi Parajuli[1] , Eleanor Flynn[1] and Mukesh Dhamala[1,2,3,4,5,*]

[1] Department of Physics and Astronomy, Georgia State University, Atlanta, GA 30303, USA
[2] Neuroscience Institute, Georgia State University, Atlanta, GA 30303, USA
[3] Center for Behavioral Neuroscience, Georgia State University, Atlanta, GA 30303, USA
[4] Center for Diagnostics and Therapeutics, Georgia State University, Atlanta, GA 30303, USA
[5] Tri-Institutional Center for Translational Research in Neuroimaging and Data Science (TReNDS), Georgia State University, Georgia Institute of Technology, and Emory University, Atlanta, GA 30303, USA
* Correspondence: mdhamala@gsu.edu

Supplementary Figure S1. Three-level comparison of theta power across stimulus clarity levels.

Violin plots showing frontal (A) and parietal (B) theta power (3-7 Hz, 0-400 ms post-stimulus) for clear (0% noise, blue), moderate (40% noise, pink), and high (55% noise, tan) stimulus conditions. Red lines indicate median; black lines indicate mean. (A) Frontal theta power exhibited a step-function pattern: clear stimuli produced significantly higher power than both moderate ($Z = 4.15$, corrected $p < 0.001$, dz = 0.81) and high noise conditions ($Z = 3.16$, corrected $p = 0.003$, dz = 0.62), while the two noise levels did not differ ($Z = -0.93$, corrected $p = 0.35$, n.s.). This step-function pattern justifies pooling the 40% and 55% noise conditions for primary analyses. (B) Parietal theta power showed no consistent clarity modulation, with moderate noise unexpectedly producing higher power than high noise ($Z = 3.57$, corrected $p = 0.001$, dz = 0.70) but no significant differences involving clear stimuli. Statistical comparisons used Wilcoxon signed-rank tests with FDR correction (Benjamini-Hochberg, $\alpha = 0.05$) across all six pairwise comparisons (3 frontal + 3 parietal). ***corrected $p < 0.001$, **corrected $p < 0.01$, n.s. = not significant. n = 23 participants. See Supplementary Table S1 for complete statistics

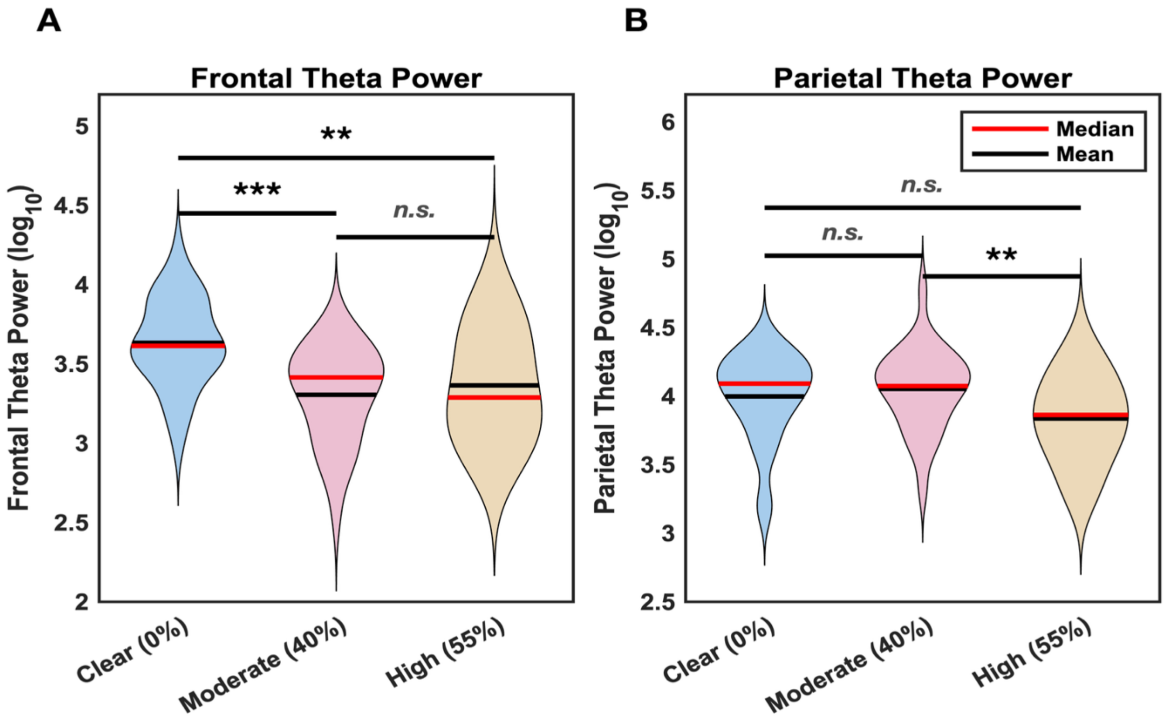

Supplementary Table S1. Pairwise comparisons of frontal and parietal theta power across three stimulus clarity levels

Region	Comparison	Z	p(uncorr)	corrected_p	dz	Sig
**Frontal**	Clear vs Moderate	4.15	<0.001	<0.001	0.81	***
**Frontal**	Clear vs High	3.16	0.002	0.003	0.62	**
**Frontal**	Moderate vs High	-0.92	0.354	0.354	-0.18	n.s.
**Parietal**	Clear vs Moderate	-0.95	0.341	0.409	-0.19	n.s.
**Parietal**	Clear vs High	2.09	0.036	0.054	0.41	n.s.
**Parietal**	Moderate vs High	3.56	<0.001	0.001	0.70	**

Note: Statistical comparisons used Wilcoxon signed-rank tests with FDR correction (Benjamini-Hochberg, $\alpha = 0.05$) across all six pairwise comparisons. Corrected p-values reflect FDR-adjusted significance levels. Effect sizes (dz) calculated as $Z/\sqrt{n}$. ***corrected $p < 0.001$, **corrected $p < 0.01$, n.s. = not significant. n = 23 participants.

Pattern: Frontal theta power exhibited a step-function pattern, with clear stimuli producing significantly higher power than both noise levels (both corrected $p \leq 0.003$), while the two noise levels did not differ from each other (corrected $p = 0.35$). This justifies pooling 40% and 55% noise levels for primary analyses while acknowledging that parietal regions showed different patterns.

Figure S2. Three-level comparison of frontoparietal imaginary coherence across stimulus clarity levels.

Frontoparietal imaginary coherence (3-7 Hz, 0-400 ms post-stimulus) for clear (0% noise, blue), moderate (40% noise, pink), and high (55% noise, tan) stimulus conditions. The connectivity pattern shows graded modulation: high noise exhibits parietal-leading phase relationships (positive iCoh, M = 0.20), while both clear (M = -0.19) and moderate (M = -0.11) noise show frontal-leading relationships (negative iCoh). Clear and moderate conditions do not differ significantly (Z = -1.13, corrected $p = 0.26$), but both differ from high noise (clear vs. high: Z = -3.39, corrected $p = 0.001$; moderate vs. high: Z = -3.57, corrected $p = 0.001$), revealing a transition from frontal-leading to parietal-leading coordination at the highest noise level. The moderate condition represents an intermediate state for connectivity, showing frontal-leading coordination like clear stimuli but distinguishable from the parietal-leading pattern at high noise. Error bars represent SEM. **corrected $p < 0.01$, n.s. = not significant. n = 23 participants. See Supplementary Table S2 for complete statistics.

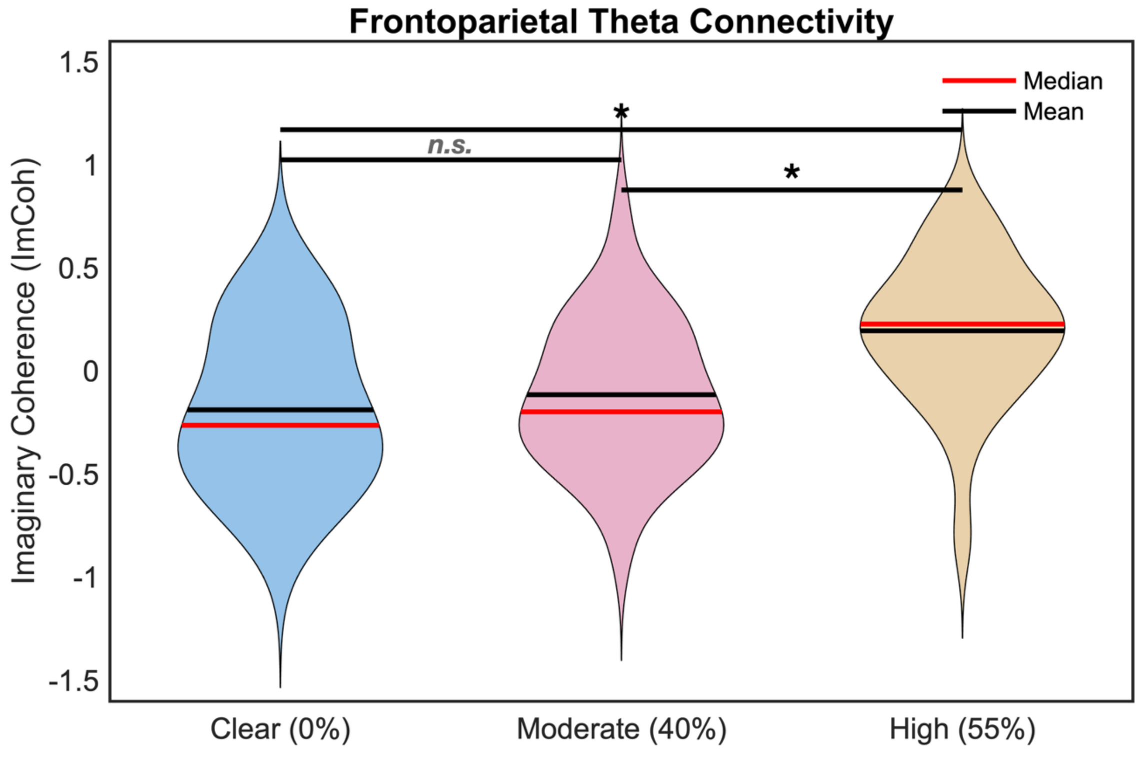

Supplementary Table S2. Pairwise comparisons of frontoparietal imaginary coherence across three stimulus clarity levels

Comparison	Z	p(uncorr)	corrected_p	dz	Sig
**Clear vs Moderate**	-1.13	0.258	0.258	-0.22	n.s.
**Clear vs High**	-3.39	<0.001	0.001	-0.66	*
**Moderate vs High**	-3.57	<0.001	0.001	-0.70	*

Note: Statistical comparisons used Wilcoxon signed-rank tests with FDR correction (Benjamini-Hochberg, $\alpha = 0.05$) across all three pairwise comparisons. Corrected p-values reflect FDR-adjusted significance levels. Effect sizes (dz) calculated as $Z/\sqrt{n}$. **corrected $p < 0.01$, n.s. = not significant. n = 23 participants.

Pattern: Frontoparietal imaginary coherence showed a graded pattern, with high noise (M = 0.20) differing significantly from both clear (M = -0.19; corrected p = 0.001) and moderate levels (M = -0.11; corrected p = 0.001), while clear and moderate did not differ (corrected p = 0.26). The moderate condition thus represents an intermediate state for connectivity, showing frontal-leading coordination like clear stimuli but distinguishable from the parietal-leading pattern at high noise. This validates pooling while acknowledging measure-specific differences, with power showing a threshold effect and connectivity showing a graded shift in phase relationships.
